# Use of anti-gSG6-P1 IgG as a serological biomarker to assess temporal exposure to *Anopheles’* mosquito bites in Lower Moshi

**DOI:** 10.1371/journal.pone.0259131

**Published:** 2021-10-27

**Authors:** Nancy A. Kassam, Neema Kulaya, Robert D. Kaaya, Christentze Schmiegelow, Christian W. Wang, Reginald A. Kavishe, Michael Alifrangis

**Affiliations:** 1 Kilimanjaro Christian Medical University College (KCMUCo), Moshi, Tanzania; 2 Centre for Medical Parasitology, Department of Immunology and Microbiology, University of Copenhagen, Copenhagen, Denmark; 3 Department of Infectious Diseases, Copenhagen University Hospital (Rigshospitalet), Copenhagen, Denmark; Instituto Rene Rachou, BRAZIL

## Abstract

**Background:**

Malaria prevalence in the highlands of Northern Tanzania is currently below 1% making this an elimination prone setting. As climate changes may facilitate increasing distribution of *Anopheles* mosquitoes in such settings, there is a need to monitor changes in risks of exposure to ensure that established control tools meet the required needs. This study explored the use of human antibodies against *gambiae* salivary gland protein 6 peptide 1 (gSG6-P1) as a biomarker of *Anopheles* exposure and assessed temporal exposure to mosquito bites in populations living in Lower Moshi, Northern Tanzania.

**Methods:**

Three cross-sectional surveys were conducted in 2019: during the dry season in March, at the end of the rainy season in June and during the dry season in September. Blood samples were collected from enrolled participants and analysed for the presence of anti-gSG6-P1 IgG. Mosquitoes were sampled from 10% of the participants’ households, quantified and identified to species level. Possible associations between gSG6-P1 seroprevalence and participants’ characteristics were determined.

**Results:**

The total number of *Anopheles* mosquitoes collected was highest during the rainy season (n = 1364) when compared to the two dry seasons (n = 360 and n = 1075, respectively). The gSG6-P1 seroprevalence increased from 18.8% during the dry season to 25.0% during the rainy season (χ^2^ = 2.66; *p* = 0.103) followed by a significant decline to 11.0% during the next dry season (χ^2^ = 12.56; *p* = 0.001). The largest number of mosquitoes were collected in one village (Oria), but the seroprevalence was significantly lower among the residents as compared to the rest of the villages (*p* = 0.039), explained by Oria having the highest number of participants owning and using bed nets. Both individual and household gSG6-P1 IgG levels had no correlation with numbers of *Anopheles* mosquitoes collected.

**Conclusion:**

Anti-gSG6-P1 IgG is a potential tool in detecting and distinguishing temporal and spatial variations in exposure to *Anopheles* mosquito bites in settings of extremely low malaria transmission where entomological tools may be obsolete. However studies with larger sample size and extensive mosquito sampling are warranted to further explore the association between this serological marker and abundance of Anopheles mosquito.

## Introduction

More than 200 million malaria cases occur globally each year of which more than 90% occur in sub-Saharan Africa (SSA) [1]. Thus, since the burden of malaria in SSA is still high, elimination of malaria seems to be a farfetched goal despite the gains achieved following scaling up of malaria control measures [2]. In Tanzania, significant declines in malaria prevalence and incidence has been reported between 2000 and 2015 in certain regions and in the country as a whole [3–6] but there has been limited progress in reducing malaria after 2015 [1]. The prevalence of malaria varies by regions from <1% in northern highlands to as high as 15% in the southern regions and 24% along the Lake and Western Zones [7]. In order to control the burden of malaria and monitor progress towards elimination, it is important to assess potential for resurgence in low malaria prevalence settings and select most efficient vector control interventions for the rest. This requires tools to measure risk of exposure and monitor if prevention of human vector contact is sufficient to control transmission.

The gold standard tool for estimating malaria transmission is to measure the entomological inoculation rate (EIR) which is the number of *Anopheles* infective bites per person per unit time, usually expressed per year [8]. This tool is however highly challenged, firstly because the procedure exposes the human sample bait to malaria infection rendering it unethical [9]. Secondly, it is expensive, difficult to apply and cumbersome as it usually involves tedious techniques such as human landing catches. Thirdly, this technique is *Anopheles* density dependent and cannot be applied in areas with low density of *Anopheles* mosquito populations [8].

Malaria transmission can also be estimated using malaria parasite exposure biomarkers through detection of antibodies against malaria parasite antigens such as AMA-1 and MSP-1 where antibody responses against malaria parasite antigens is an effective proxy of the level of exposure to malaria parasites [10, 11]. But malaria parasite antigens may not be suitable to monitor and measure risk for malaria transmission in areas where there is close to no transmission as they last for a short time in absence of re-exposure [12] and may be negative for most individuals due to low malaria prevalence. Alternatively, determining the level of human antibodies against specific *Anopheles* mosquito salivary antigens provide another attractive proxy of vector exposure and thus, potential transmission [13]. When an *Anopheles* mosquito takes a blood meal, it injects saliva containing proteins. Some of these proteins are functional and necessary for blood uptake as they prevent blood coagulation. The mosquito salivary proteins are also antigenic and will stimulate an immunological response causing the host immune cells to produce antibodies against each specific immunogenic salivary protein [14]. The antibodies produced can be serologically detected and quantified reflecting the level of exposure to *Anopheles* bites independent of any malaria parasite infection [15, 16]. The antibodies may act as an effective tool for estimation of *Anopheles* mosquito increase as a proxy for increased risk of malaria transmission [17]. Moreover, compared to EIR, these antibody assessments are easier to apply [13], more sensitive for detection of exposure to vector bites [18] and more ethically convenient.

Several *Anopheles* mosquito salivary proteins have been identified using transcriptome analyses [19, 20] and evaluated as proxies for exposure to *Anopheles* mosquito bites [14]. In particular one protein, the *gambiae* Salivary Gland protein 6 Peptide 1 (gSG6-P1) have been shown to be unique to *Anopheles* mosquitoes and is highly conserved in Afro-tropical malaria vectors; *An*. *gambiae*, *An*. *funestus* and *An*. *arabiensis* [14].

Human antibodies against gSG6-P1, detected in *Anopheles* exposed individuals, have been shown to correlate well with levels of exposure to *Anopheles* mosquitoes [15–17] and are always detected in malaria infected individuals [13]. Levels of gSG6-P1 antibodies against the antigen have shown individual and population differences correlating well with levels of exposure to *Anopheles* [15, 17, 21]. However, previous evaluations compared spatial exposure to *Anopheles* mosquitoes across altitude transects [15, 16] and except for a study performed among Senegalese children, showing promise in measuring anti-gSG6-P1 as a proxy for *Anopheles* exposure in a low transmission setting [18], previous studies mainly focused on areas with moderate to high malaria transmission [15, 16, 22–24]. As assessment of gSG6-P1 antibody levels may also be used to determine efficacy of different interventions such as measures used for vector control [25, 26], a prerequisite is that the estimation of gSG6-P1 antibodies is well measurable in areas with very low transmission.

Furthermore, the previous evaluations of the validity of measuring gSG6-P1 as a proxy of *Anopheles* exposure were largely carried out among children [15, 17, 18, 21, 27, 28]. The association between age and exposure to *Anopheles* mosquitoes is nevertheless important to evaluate across different age groups in order to help direct interventions to the most vulnerable age groups. The current study aimed to evaluate the use of anti-gSG6-P1 IgG antibodies as a tool to detect exposure to *Anopheles* mosquito bites, discriminate spatial and temporal variation in exposure and determine possible associations between vector exposure and socio-demographic characteristics in a setting of Lower Moshi, Tanzania which is a low malaria endemic area earmarked for malaria pre-elimination.

## Materials and methods

### Study site

**Fig 1:**
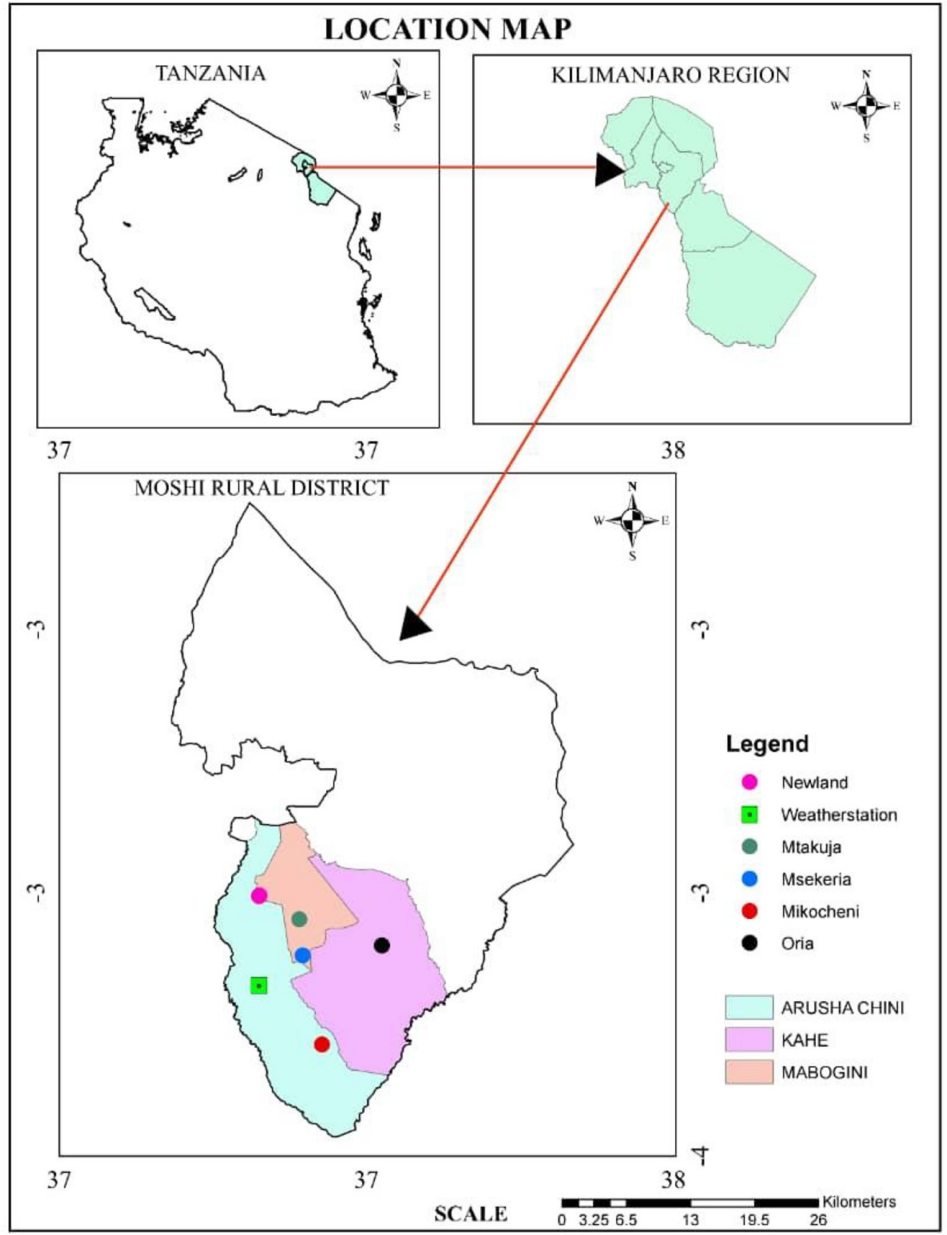
Location of Lower Moshi within Kilimanjaro District, Tanzania.

The study site of Lower Moshi (latitude 3°61’-3°68’S; longitude 37°32’-37°38’E), is located 10 kilometres from Moshi municipality, and about 800 meters above sea level in rural Moshi, south of Mount Kilimanjaro, northern-eastern Tanzania (Fig 1) (ArcGIS version 10.4, Esri). Lower Moshi area includes three wards namely Kahe, Arusha Chini and Mabogini.

Most of the population in the area is engaged in agricultural activities with irrigated rice and sugarcane cultivation as main crops. The non-irrigated crops include maize, beans and banana. Two rivers, namely Njoro and Rau provide the water for irrigation. Livestock in this area are mainly cattle, goats, sheep and poultry [29].

Transmission of malaria occurs throughout the year with low parasitemia and prevalence less than 0.1% [29] with an EIR of 0.54 [30] and human biting index for *An*. *arabiensis* ranges between 0.1 and 0.3 for outdoor and 0.4 and 0.9 for indoor mosquitoes [31]. The yearly proportions of malaria cases reported at the local Tanganyika Plantation Company (TPC) hospital is low, shown to fluctuate between 0.5% - 2.3% in a ten-year period from 2009–2018 [32]. Fluctuation in mosquito prevalence between seasons is observed and in particular for *An*. *arabiensis*, the predominant malaria vector in the area where irrigation activities provide important breeding sites [31].

### Study design and sampling procedures

We conducted three cross sectional surveys in the study area in 2019. The baseline survey was conducted during the dry season (March) followed by two follow-up surveys during the end of the rainy season (June) and during dry season (September).

Participants for the surveys were identified through a multistage sampling technique consisting of three stages which were independent of the three surveys. Individuals aged six months and above were recruited from five selected villages in Lower Moshi. At stage one, five villages surrounding TPC, a sugar factory in Lower Moshi, were selected: Oria village located close to rice paddies; Mtakuja, Mserekia and Newland villages surrounded by sugarcane plantations and Mikocheni village which is mostly located within the savannah grasslands. At stage two, approximately 600 households were visited to assess their willingness to participate in the surveys, from which 229 households (ranging 25 to 60 households per village) were randomly selected. At stage three, a maximum of four members per household with different sex and age were invited to participate in the surveys. Parents and guardians of children aged less than 18 years were also requested to assist their selected children’s participation. Participants were issued identification tags matching the identification details in their record forms to ensure participation of the same participant in all three surveys.

### Ethical approval

Written informed consent and/or ascent was obtained from the adult participants while children’s guardians or parents were asked to give the consent. Approval to conduct the surveys was provided by Kilimanjaro Christian Medical University College (KCMUCo) Research Ethics and Review Committee (CRERC). Permission to carry out the study in Lower Moshi was sought from the District Executive Director (DED) of Moshi District Council and local government leaders of Lower Moshi.

### Data collection

At the baseline survey, we performed face-to-face interviews for enrolled participants using questionnaires, tested them for malaria using malaria rapid diagnostic test (mRDT) (SD-BioLine, Standard Diagnostics, INC, Korea) and collected approximately 500 μL of blood sample in EDTA containing tubes during initial contact. Data was collected electronically using Open Data Kit (ODK) application (ODK collect version 1.30.1; link: https://odk-collect.en.uptodown.com/android). Data on socio-demographic characteristics of the participants including age, sex and village of residence were collected. Also, data regarding bed net ownership, use, impregnation with insecticides, presence of holes and the size of holes were collected. During the second and third visits, the same procedure was repeated except for the interviews. A CDC miniature light trap for collection of mosquitoes was set at a systematic interval after every eight households.

### Mosquito and rainfall data collection

At each visit, mosquitoes were collected from a total of 32 households using CDC miniature light traps for one-night following collection of blood sample. A trap was hung in the participant’s room at the feet side of the bed, at approximately 1 meter from the floor.

Rainfall data was provided by TPC Sugar Factory located at the centre of the villages selected for the study. Daily rainfall data issued was recorded in millimetres of rain at the factory’s meteorological station from January 2019 to January 2020.

### gSG6-P1 ELISA

Plasma was isolated from whole blood samples at 1500 x*g* for 5 minutes and stored at -80°C until use. Synthetic gSG6-P1, the antigen (Catalogue number 2958–003 Genepep, Saint Jean de Vedas-France) was dissolved in ultra-filtered water to a final working concentration of 10 μg/mL.

Enzyme Linked Immuno-Sorbent Assay (ELISA) technique was performed as described elsewhere [16]. Briefly, ELISA plates (Sero-Well, Sterilin Appleton Woods Limited) were coated with gSG6-P1 antigen and incubated. Plates were blocked using Qiagen blocking buffer (Qiagen, Penta-His Conjugate kit) then 20% plasma was added followed by overnight incubation at 2–8⁰C. To detect bound human anti-gSG6-P1 IgG, goat anti-human IgG horseradish peroxidase (HRP) conjugated antibody (Thermo Fisher Scientific) was added. 2,2’-Azinobis (3-ethylbenzothiazoline-6-sulfonic acid) (ABTS) (Roche, Germany) substrate was added and the reaction was stopped using 20% sodium dodecyl sulphate (SDS) (Sigma-Aldrich) solution. Optical densities (ODs) were read at 405 nm using Multi-Scan FC microplate photometer (Thermo Scientific, Life Technologies Corporation) ELISA reader and the final ODs of each sample was obtained as ΔODs by finding the average of ODs from two antigen-coated wells subtracted the OD obtained from an uncoated well. Cut-offs for seropositivity were determined per plate as mean ΔODs of negative controls plus two standard deviations.

For quality control, one positive control plasma sample obtained from confirmed malaria positive cases and negative control plasma samples donated from seven Danish volunteers were included in each run as cut-offs were determined per every run. All positives were re-run in one plate with five negative samples picked at random to confirm results.

### Mosquito species identification

Mosquitoes collected from households were sorted according to their genus and counted. *Anopheles* mosquitoes were processed to obtain tissues for Deoxy-ribonucleic Acid (DNA) extraction using the Chelex-100 method as previously described [33]. Extracted DNA concentrations were measured in ng/μL using NanoDrop One^c^ (Thermo scientific).

As previously described [34], Polymerase Chain Reaction (PCR) TaqMan assay for *Anopheles gambiae s*.*l*. sibling species identification was used to identify *An*. *gambiae* s.s., *An*. *arabiensis* and the non-vector sibling *An*. *gambiae s*.*l*. species to species level as they cannot be morphologically distinguished. The non-vector An. gambie s.l. species including *An*. *melas*, *An*. *merus* and *An*. *quadrianulatus* were identified as a group.

PCR was performed in a reaction volume of 10 μL containing 3 μL distilled water, 5 μL Sensi-mix reaction buffer (Bioline, Meridian Bioscience), 0.5 μL primer and probe mix (Applied Biosystems–Thermo Fisher Scientific), 0.5 μL LNA probe (Sigma-Aldrich) and 1 μL Chelex extracted genomic DNA in 200 μL optical caped PCR reaction tubes (Greiner Bio-One).

Positive control DNA for *An*. *gambiae* s.s DNA, *An*. *quadriannulatus* DNA and *An*. *arabiensis* and a negative control (distilled water) were included in every test. PCR was done using Stratagene Mx 3005P real time thermocycler (Agilent Technologies, Santa Clara, California) at a standard 45 cycles thermo profile of initial activation at 95°C for a single 10 minutes cycle, template denaturation at 95°C for 45 cycles for 25 seconds, annealing and elongation at 67°C for 45 cycles for 45 seconds.

### Statistical analysis

Data were analysed using Stata Version 14 (StataCorp, Texas, USA) and GraphPad Prism version 9 (San Diego, California, USA) softwares. Chi square (χ^2^) was used to compare temporal variations in anti-gSG6-P1 seroprevalence for all three surveys. The association between gSG6-P1 seroprevalence and socio-demographic characteristics and bed net ownership, use, and status were determined using both univariate and multivariate logistic regression analyses. Multivariate logistic regression was performed with inclusion of all variables with *p* < 0.2 in the univariate model. All differences were regarded statistically significant at *p* values < 0.05.

### Variables

The independent variables included village of residence, age, sex, education level including individuals who had primary, secondary, tertiary education and those who never had formal education, bed net ownership, bed net daily usage, bed net having holes and size of holes on bed nets. The dependent variable was “gSG6-P1 seropositivity” defined as anti-gSG6-P1 IgG levels above the negative value cut-offs.

## Results

### Study population

In total, 308 study participants were enrolled during the baseline survey. Of them, 201 (65.3%) were followed in the second survey and 204 (66.2%) in the third survey (Table 1). Absent participants during the follow-up studies had either travelled or absent for other community and employment activities. Children aged between 6 and 15 years represented the largest age-group, whereby 77 (25.0%) participated in the first, 50 (24.9%) in the second and 64 (31.4%) in the third survey. Almost 70.0% of participants in each survey were females as men were out in the fields working or away for other community and employment activities. Slightly less than 40% of participants in all three surveys had primary education. Malaria prevalence was below 0.5% across surveys where the number of positive participants were 1, 0 and 1 during the first, second and third survey, respectively.

**Table 1:** Socio-demographic characteristics of the study population and malaria positivity by survey: N = 308.

	Survey 1 (N = 308)	Survey 2 (N = 201)	Survey 3 (N = 204)
Variable	n (%)	n (%)	n (%)
***Village of residence*:**			
Oria	52 (16.9)	43 (21.4)	40 (19.6)
Mtakuja	40 (13.0)	35 (17.4)	34 (16.7)
Newland	49 (15.9)	28 (13.9)	25 (12.3)
Mikocheni	84 (27.3)	44 (21.9)	54 (26.5)
Mserekia	83 (27.0)	51 (25.4)	51(25.0)
***Age of participants (years)*:**			
0–5	49 (15.9)	32 (15.9)	32 (15.7)
6–15	77 (25.0)	50 (24.9)	64 (31.4)
16–30	33 (12.0)	18 (9.0)	16 (7.8)
31–45	50 (16.2)	31 (15.4)	28 (13.7)
46–65	60 (19.4)	43 (21.4)	41 (20.1)
66+	35 (11.4)	27 (13.4)	23 (11.3)
Median (Range)	28 (0.7–94)	31 (0.8–86)	21 (0.8–86)
***Participants’ sex*:**			
Female	215 (69.8)	136 (67.7)	140 (68.6)
Male	93 (30.2)	65 (32.3)	64 (31.4)
***Education level from children enrolled in schools and above***	*(n = 271)*	*(n = 176)*	*(n = 178)*
No formal education	56 (18.2)	35 (17.4)	31 (15.2)
Pupils at primary school	76 (24.7)	52 (25.9)	64 (31.4)
Had primary education	119 (38.6)	79 (39.3)	74 (36.3)
Secondary education	17 (5.5)	7 (3.5)	6 (2.9)
Tertiary education	3 (1.0)	3 (1.5)	3 (1.5)
***Malaria prevalence by mRDT***			
Positive	1 (0.3)	0 (0.0)	1 (0.5)
Negative	307 (99.7)	201 (100.0)	203 (99.5)

### Baseline characteristics of the study population by village of residence

The baseline characteristics of the study population by village of residence are shown in Table 2. Most of the characteristics including age, education level, bed net ownership, bed net daily usage, impregnation with insecticides and numbers of bed nets having holes varied significantly across villages (*p*<0.05). In all five villages, there were more female than male participants and most of the participants had some level of formal education (primary education and above). More than 60% of participants from all villages owned bed nets and more than 50% of participants used bed nets. Oria village had the highest number of participants owning and using bed nets (94.2% and 92.3%, respectively).

**Table 2:** Baseline characteristics of the study population by village of residence: N = 308.

Village	Oria n = 52	Mtakuja n = 40	Newland n = 49	Mikocheni n = 84	Mserekia n = 83	*p*-value
**Age**						
0–15	18(34.2)	17 (42.5)	16 (32.7)	50 (59.5)	25 (30.1)	
16–45	16(30.8)	8 (20.0)	22 (44.9)	23 (27.4)	18 (21.7)	
46+	18 (34.6)	15 (37.5)	11 (22.5)	11 (13.1)	40 (48.2)	0.001
**Sex**						
Female	31 (59.6)	31 (77.5)	31 (63.3)	61 (72.6)	61 (73.5)	
Male	21 (40.4)	9 (22.5)	18 (36.7)	23 (27.4)	22 (26.5)	0.237
**Education level n = 271**	***n = 47***	***n = 35***	***n = 43***	***n = 76***	***n = 70***	
No formal education	1 (2.1)	5 (14.3)	5 (11.6)	16 (21.1)	29 (41.4)	
Pupils at primary school	13 (27.7)	8 (22.9)	7 (16.3)	37 (48.7)	11 (15.7)	
Primary education and above	33 (70.2)	22 (62.9)	31 (72.1)	23 (30.3)	30 (42.9	0.001
**Bed net ownership**						
No	3 (5.8)	8 (20.0)	5 (10.2)	14 (16.7)	31 (37.4)	
Yes	49 (94.2)	32 (80.0)	44 (83.3)	70 (83.3)	52 (62.6)	0.001
**Bed net daily usage**						
No	4 (7.7)	10 (25.0)	14 (28.6)	27 (32.1)	37 (44.6)	
Yes	48 (92.3)	30 (75.0)	35 (71.4)	57 (67.9)	46 (55.4)	0.001
**Bed net impregnation n = 247**	***n = 49***	***n = 32***	***n = 44***	***n = 70***	***n = 52***	
No impregnation	17 (34.7)	2 (6.3)	10 (22.7)	24 (34.3)	20 (38.5)	
Insecticide impregnated	32 (65.3)	30 (93.8)	34 (77.3)	46 (65.7)	32 (61.5)	0.013
**Bed net having holes n = 247**	***n = 49***	***n = 32***	***n = 44***	***n = 70***	***n = 52***	
No holes	21 (42.9)	13 (40.6)	36 (81.8)	38 (54.3)	13 (25.0)	
Bed nets with holes	28 (57.1)	19 (59.4)	8 (18.2)	32 (45.7)	39 (57.0)	0.001
**Size of holes n = 127**	***n = 29***	***n = 19***	***n = 8***	***n = 32***	***n = 39***	
Very small-small	7 (24.1)	8 (42.1)	4 (50.0)	14 (43.8)	16 (41.0)	
Medium	14 (48.3)	6 (31.6)	1 (12.5)	8 (25.0)	14 (23.1)	
Large-very large	8 (27.6)	5 (26.3)	3 (37.5)	10 (31.3)	9 (23.1)	0.581

### Rainfall pattern and *Anopheles* mosquito abundance

**Fig 2:**
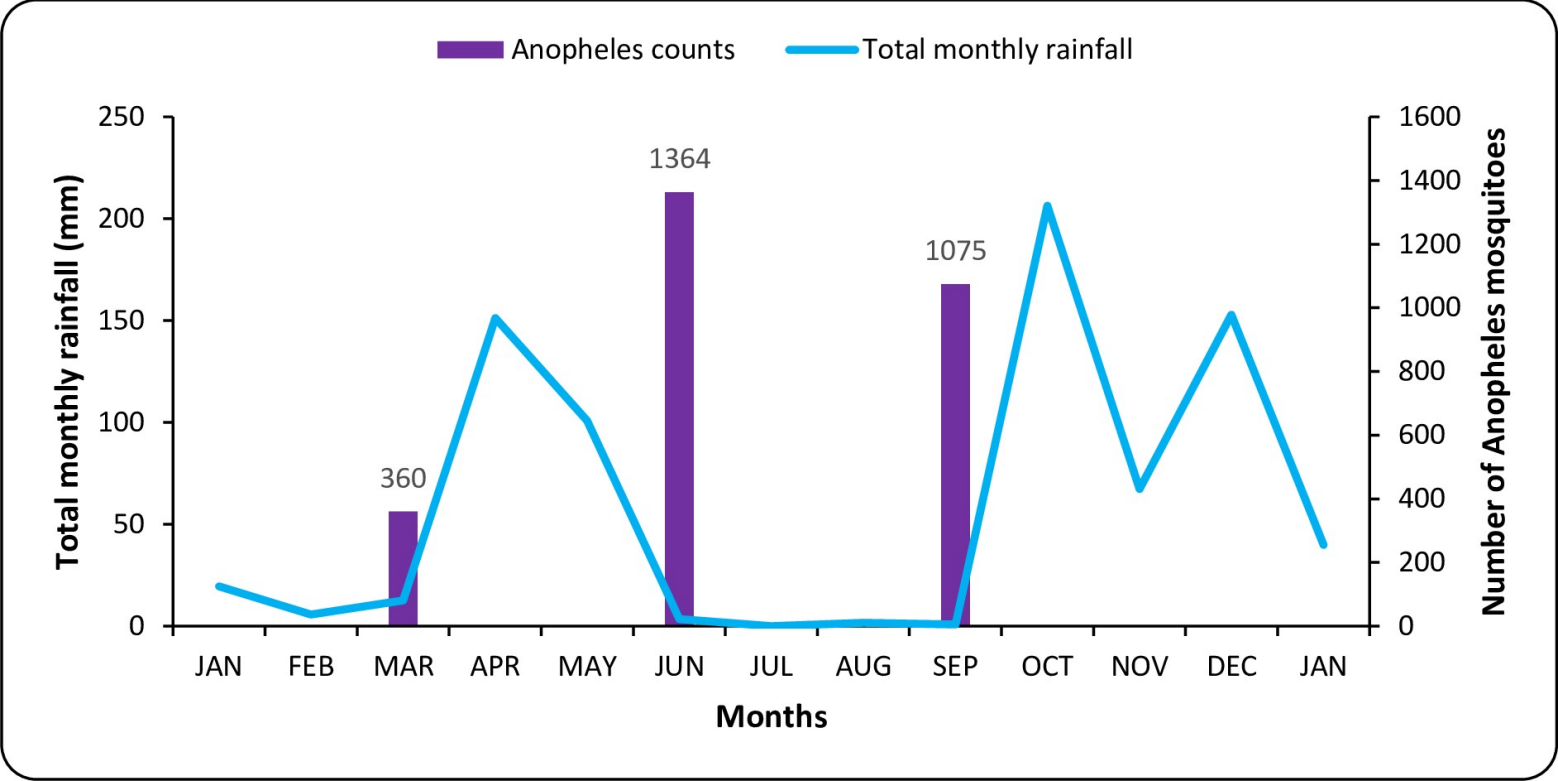
*Anopheles* mosquitoes collected in all villages (32 households) at three time points and total monthly rainfall for Lower Moshi.

Monthly rainfall pattern for Lower Moshi in the year 2019 is shown in Fig 2. The rains were seen from April until June then followed a three months period of drought from June to September, after which began another rainy season. 2,799 *Anopheles* mosquitoes were collected from 32 households within the selected villages during the three cross sectional surveys (S1 Table). *Anopheles* mosquito density was lowest during the first survey (n = 360) conducted in March when it was dry and increased during the second survey (n = 1364) conducted at the end of the rainy season followed by another slight decline at the third survey in September (n = 1075).

### Distribution of *Anopheles* mosquitoes in the study villages

Table 3 shows numbers of mosquitoes collected in the study villages at each survey. Generally, there were more mosquitoes collected during the second survey than at the first and the third surveys and as compared to the other villages, Oria village had by far the highest number of sampled mosquitoes; 338, 1117 and 998 at survey 1, 2 and 3, respectively (Table 3). Of the 2,799 *Anopheles* mosquitoes collected, DNA samples from 1,012 of these were analysed by PCR to determine sibling species out of which, 1,009 (99.7%) gave conclusive results. *An*. *arabiensis* was the most dominant species (99.4%) and only six (0.6%) mosquitoes were identified as other *An*. *gambiae s*.*l*. species (*An*. *melus*, *An*. *merus or An*. *quadrianulatus*) and there was no difference in sibling species between the three cross sectional studies from all three surveys.

**Table 3:** *Anopheles* mosquitoes collected in each village.

	Survey 1	Survey 2	Survey 3	Total
Oria	338	1117	998	2453
Mtakuja	7	62	33	102
Newland	0	16	27	43
Mikocheni	14	58	0	72
Mserekia	1	111	17	129
**Total**	**360**	**1364**	**1075**	**2799**

### Temporal variation in gSG6-P1 seroprevalence in the study villages

**Fig 3:**
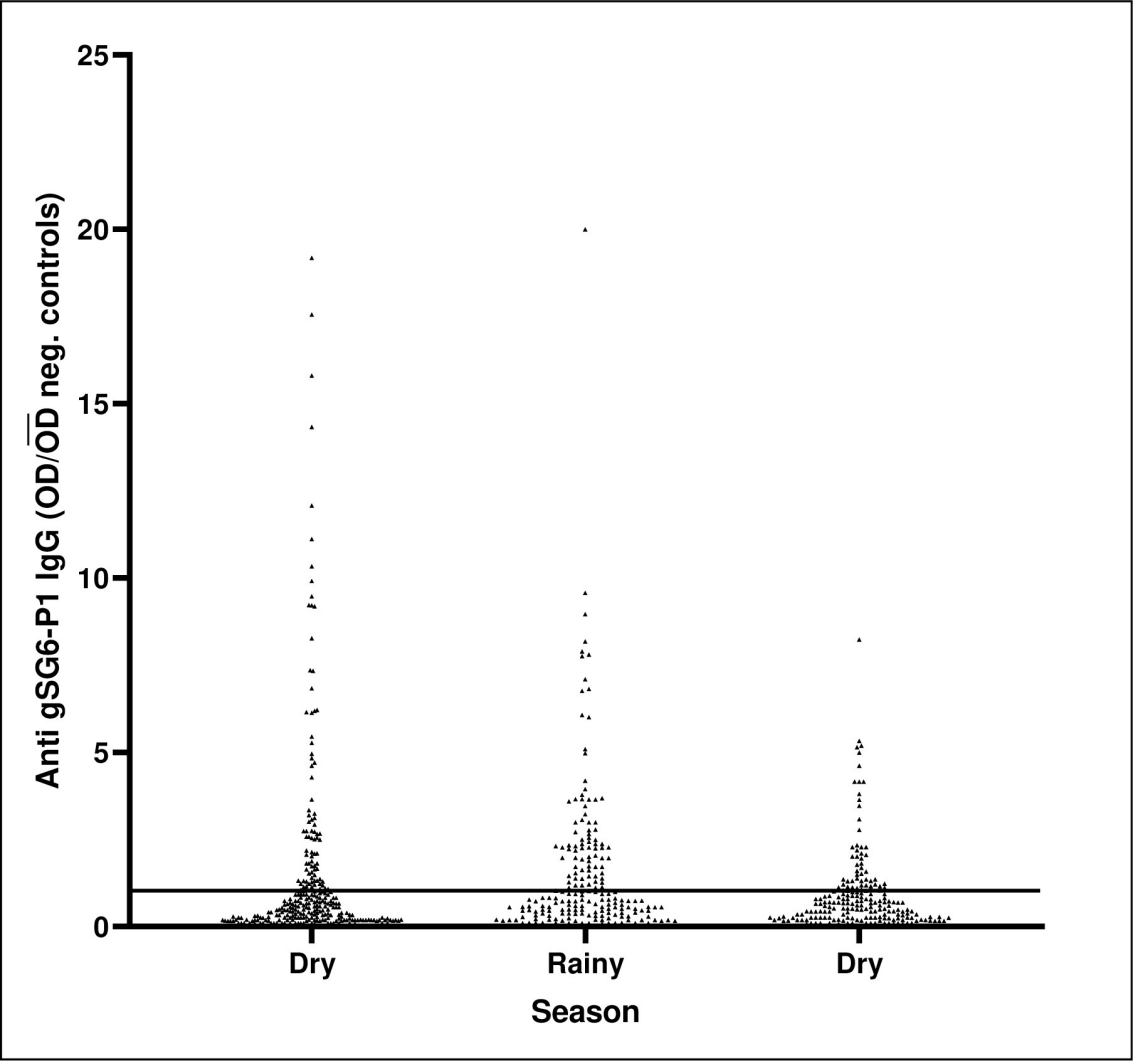
Individual anti-gSG6-P1 IgG levels measured during the dry season in March, rainy season in June and another dry season in September 2019; the bar at 1 shows the mean OD of negative controls.

The gSG6-P1 seroprevalence was 18.8% (58/308) during the first survey conducted in all villages during dry season with low mosquito abundance. The seroprevalence increased to 25.0% (50/201) at the second survey (χ^2^ = 2.66; *p* = 0.103) conducted at the end of the rainy season with increased *Anopheles* mosquito abundance and then followed by a significant decline to 11.0% (23/204) at the third survey (χ^2^ = 12.56; *p* = 0.001) conducted during the subsequent dry season with lower *Anopheles* mosquito density (Fig 3).

**Fig 4:**
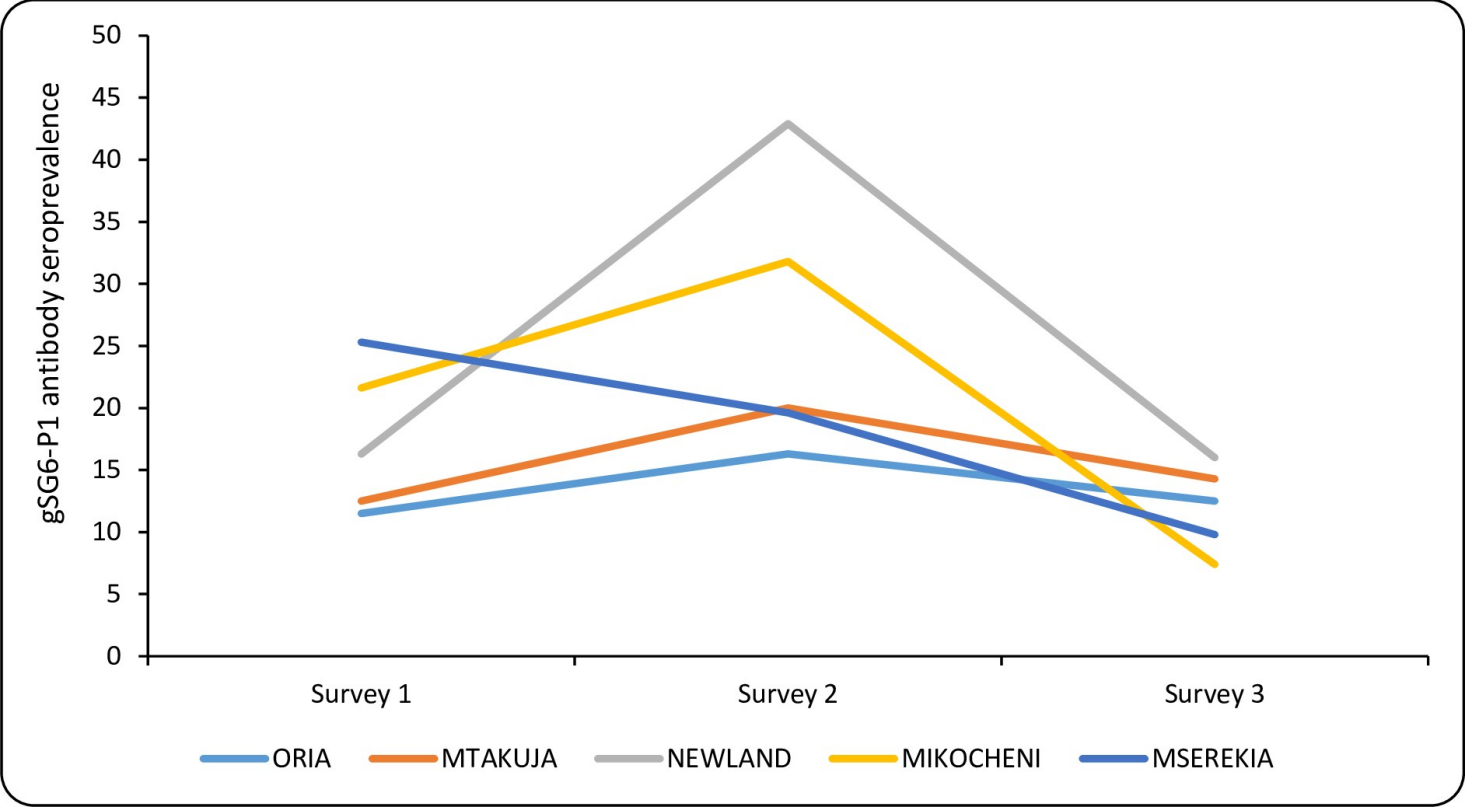
Temporal variation of gSG6-P1 seroprevalence for the five villages included in the study during the three surveys.

The variation in gSG6-P1 seroprevalence at surveys 1, 2 and 3 shown for each of the villages included in the study is shown in Fig 4. The seroprevalence pattern was similar for all (except Mserekia), with a peak during the second survey, which was also the time where the largest differences in mean gSG6-P1 seroprevalence between villages was seen ranging between 16.3% (Oria) and 42.9% (Newland). During the first survey the range was between 11.5 and 25.3%, and during the third; 7.4 and 16%. During the second survey, sero-prevalence was significantly lower for Oria residents as compared to the other villages (*p* = 0.039). There was no statistical significance in seroprevalence variation by village of residence at survey 1 and 3.

### Individual gSG6-P1 IgG levels and numbers of *Anopheles* mosquitoes collected

For fifty four individuals’ gSG6-P1 IgG levels were compared to the numbers of *Anopheles* mosquitoes collected in their respective homes (S1 Fig). There were no statistically significant correlations between individual levels of gSG6-P1 IgG and numbers of *Anopheles* mosquitoes collected in all three surveys (Pearson correlation coefficient (r) = -0.037, *p* = 0.7912; r = -0.034, *p* = 0.8166; r = 0.0323, *p* = 0.8303 for survey 1, 2 and 3 respectively).

### Factors associated with risk for exposure to *Anopheles* bites using anti-gSG6-P1 IgG levels as proxy for exposure

The associations between anti-gSG6-P1 antibody seropositivity and village of residence, demographic characteristics, bed net ownership, use and condition of the bed nets were explored by logistic regression (Table 4). In the univariate analysis, risk of exposure to *Anopheles* bites were significantly lower among residents of Oria village (OR = 0.26; 95% CI = 0.09–0.78; *p* = 0.016) and for Mserekia village residents (OR = 0.33; 95% CI = 0.12–0.90; *p* = 0.031) as compared to Newland residents during the rainy season (survey 2). The risk of exposure to *Anopheles* bites were not of statistical significance by villages of residence during the dry seasons. During all three surveys, risk of exposure to *Anopheles* bites had no significant associations with age, sex, education level, bed net ownership, usage, insecticide impregnation, presence of holes and size of holes. In the multivariate logistic regression analysis, however; only participants living in Oria village still had a significantly lower risk of exposure to *Anopheles* bites as compared to the rest of the villages (AOR = 0.29; CI = 0.09–0.94; *p* = 0.039) while exposure to *Anopheles* mosquito bites was not significantly associated with any of the other variables.

**Table 4:** Univariate and multivariate analysis of factors associated with risk of exposure to *Anopheles* bites in Lower Moshi.

Variable	Survey 1				Survey 2				Survey 3			
	COR (95% CI)	*p*-value	AOR (95% CI)	*p*-value	COR (95% CI)	*p*-value	AOR (95% CI)	*p*-value	COR (95% CI)	*p*-value	AOR (95% CI)	*p*-value
**Village**												
Newland	1				1		1		1			
Oria	0.67 (0.21–2.09)	0.488			0.26 (0.09–0.78)	0.016	0.29 (0.09–0.94)	0.039	0.75 (0.18–3.11)	0.692		
Mtakuja	0.73 (0.22–2.44)	0.612			0.33 (0.11–1.02)	0.054	0.45 (0.14–1.50)	0.195	0.91 (0.22–3.78)	0.891		
Mikocheni	1.40 (0.56–3.51)	0.475			0.62 (0.23–1.66)	0.343	0.59 (0.20–1.76)	0.343	0.42 (0.10–1.84)	0.250		
Mserekia	1.74 (0.70–4.29)	0.232			0.33 (0.12–0.90)	0.031	0.39 (0.12–1.22)	0.105	0.57 (0.14–2.34)	0.436		
**Age categories**												
0–15	1		1		1		1		1		1	
16–45	1.68 (0.84–3.38)	0.145	1.68 (0.84–3.38)	0.145	1.03 (0.47–2.25)	0.949	1.84 (0.17–19.60)	0.613	0.71 (0.18–2.75)	0.617	0.67 (0.17–2.63)	0.567
46+	1.68 (0.84–3.38)	0.339	1.68 (0.84–3.38)	0.339	0.59 (0.27–1.27)	0.173	1.12 (0.11–11.57)	0.926	2.01 (0.78–5.16)	0.149	1.93 (0.75–5.01)	0.175
**Sex**												
Female	1				1				1			
Male	0.30 (0.51–1.78)	0.871			0.98 (0.49–1.94)	0.953			0.75 (0.28–2.00)	0.563		
**Education level**												
No formal education	1				1		1		1			
Pupils at primary school	0.98 (0.42–2.27)	0.958			2.12 (0.77–5.79)	0.144	2.58 (0.24–27.77)	0.435	1.15 (0.28–4.77)	0.851		
Had primary education and above	0.88 (0.42–1.90)	0.752			1.24 (0.47–3.23)	0.667	1.09 (0.38–3.14)	0.879	1.43 (0.37–5.50)	0.606		
Bed net ownership*												
No	1				1				1		1	
Yes	0.93 (0.46–1.90)	0.851			0.63 (0.29–1.37)	0.242			4.73 (0.6–36.3)	0.136	4.68 (0.60–36.21)	0.139
**Bed net daily use**												
No	1				1				1			
Yes	0.76 (0.30–1.88)	0.546			0.55 (0.21–1.49)	0.242			1.10 (0.3–4.0)	0.889		
**Impregnation**												
No	1				1				1			
Yes	0.84 (0.42–1.67)	0.615			0.68 (0.31–1.49)	0.336			1.35 (0.5–3.7)	0.559		
**Bed net having holes**												
No	1				1				1			
Yes	0.77 (0.40–1.46)	0.421			0.84 (0.4–1.8)	0.640			0.66 (0.3–1.6)	0.357		
Size of holes*												
Very small-Small	1				1				1			
Medium	1.17 (0.37–3.64)	0.791			1.00 (0.3–3.2)	1.00			1.18 (0.2–7.6)	0.860		
Large-very large	1.50 (0.47–4.74)	0.490			1.40 (0.4–4.6)	0.51			2.52 (0.5–13.5)	0.283		

COR–crude odds ratios; AOR–adjusted odds ratios; CI–confidence interval.

### gSG6-P1 seroprevalence by daily bed net use

The seroprevalence by bed net use are shown in S2 Fig. There was no statistically significant variation in gSG6-P1 seroprevalence by daily bed net usage within different age groups, although seroprevalence was clearly lower (6%) among individuals using bed nets compared to individuals who did not use bed nets (13%) for children aged between 0 and 5 years. Individuals aged 46 years and above reported more daily use of bed nets when compared to the rest of the age groups (75.8%) while children aged between 6 and 15 years reported least use of bed net when compared to the rest of the groups (59.7%) with similar gSG6-P1 seroprevalence among those who used bed nets and those who did not use bed nets.

## Discussion

This study evaluated human antibody responses to the *gambiae* Salivary Gland protein 6 Peptide 1 (gSG6-P1) as a serological biomarker of *Anopheles* exposure in a malaria elimination-prone setting with very low malaria transmission intensity. The serological responses were measured against *Anopheles* mosquito density by spatial and temporal assessment and as well explored in terms of possible associations to characteristics of the enrolled participants.

This study sampled a total of 2,799 *Anopheles* mosquitoes from the five villages and a temporal variation for the three surveys was observed. Out of these, 1,012 were analysed for species identification and more than 99% were identified as *An*. *arabiensis*. Several other studies have also reported that *An*. *arabiensis* is the most dominant *Anopheles* mosquito species in the same setting [31, 35, 36] and the predominance of *An*. *arabiensis* in this setting is probably due to changes in the composition of *Anopheles* sibling species over time [37].

The largest numbers of mosquitoes were by far collected in Oria village likely due to large fields of rice paddies and thick vegetation located proximal to the households of this village, while the four other villages, which are either proximal to the sugarcane plantations or within the savannah grasslands, had significantly lower numbers of *Anopheles* mosquitoes.

In this study, significant correlations between individual or household levels of exposure and numbers of *Anopheles* mosquitoes collected were not found. High gSG6-P1 IgG titres were detected from individuals and households from which low numbers of *Anopheles* mosquitoes were collected and vice versa. Several other studies have observed lack of correlations between gSG6-P1 antibodies and entomological indices including human biting rate [17, 22, 38] and human landing catches [39]. Contrary to these findings, a study conducted in Korogwe Northern Tanzania found significant correlations between numbers of *Anopheles* mosquitoes collected and household levels of exposure to *Anopheles* bites [15]. The differences seen between that particular study and the current study are most likely due to the differences in transmission intensity and the dominant *Anopheles* mosquitoes. Lower Moshi is regarded a low transmission intensity area and the lack of association between mosquito abundance and exposure is probably due to very few to zero mosquitoes caught in some of the villages especially during the two dry seasons and this finding is supported by studies which have shown that use of entomological tools to estimate malaria risk could be challenging and less useful [40]. On the other hand, Korogwe is an area of moderate malaria transmission intensity with stable malaria heterogeneity hotspots [15]. Also, in Lower Moshi, *An*. *arabiensis* is the dominant vector while in Korogwe, the dominant vectors are mainly a mixture of *An*. *gambiae s*.*l*. (80%), and *An*. *funestus* (18.6%) [15]. Different mosquito behaviours affect host exposure regarding when, where and how much an individual gets exposed.

Temporal variations in gSG6-P1 seroprevalence were seen in our study during the three surveys and as expected, the response increased during the rainy season along with an increased number of *Anopheles* mosquitoes collected during this season. These finding are similar to previous studies findings where gSG6-P1 seroprevalence varied significantly with seasons and were higher during high malaria transmission seasons both in the low malaria transmission settings and high malaria transmission settings [16, 17, 26]. Despite the observed seasonal variation, the sero-prevalence was unexpectedly lower during the second dry season when more *Anopheles* mosquitoes were collected when compared to the first dry season. This suggests that gSG6-P1 seroprevalence is not reflected by mosquito density as supported by several other studies [17, 22, 38, 39].

Generally, Oria village showed the lowest prevalence of anti-gSG6-P1 antibodies during our study period and thus, presumably reflecting that a low number of individuals are directly exposed to *Anopheles* bites despite the higher numbers of *Anopheles* mosquitoes collected on average in this particular village than other villages. This is likely due to more proper utilization of bed nets in this village due to high mosquito density. In agreement with this observation, bed net ownership and daily usage were explored and while in Oria village, 94.2% of participants owned bed nets and 92.3% of participants confirmed daily usage of bed nets, the usage was only between 55.4%-75.0% in the other villages. Our findings are supported by another study done among a rice farming community in Kenya, where protection against mosquito bites was the main reason for using a bed net (95%) followed by protection against malaria infection (54%) [41]. However, whether bed nets were treated with insecticides differed markedly between the villages and in Oria village in particular only 65% of the bed nets were treated. Thus, the actual biting rate for Oria is expected to be higher than other villages, yet the observed sero-prevalence of exposure was lower. This observation is similar to what was reported by another study also done in Lower Moshi, which found that the risk of malaria infection based on EIR was 61–68% less for people living in villages close to rice fields compared to villages surrounded by sugarcane plantations or savannah [35]. This study reported that rice fields were a major source of the *An*. *gambiae* complex and especially *An*. *Arabiensis*, but unexpectedly, the Human Biting Index (HBI) was significantly lower in the rice field villages likely due to more strict implementation of protective measures in proportion to biting density [35]. Further exploration on human and mosquito interaction with extensive mosquito collections are needed for the explanation behind this apparent paradox.

The overall pattern of temporal variation in gSG6-P1 seroprevalence was apparent; lower exposure during the dry seasons and increased exposure during the rainy season with the exception of residents of Mserekia during the first dry season. There was no statistically significant difference in the variation of exposure by village during the first and the third surveys, perhaps due to all villages being located within more or less similar altitudinal range. The slight non-significant variations in levels of exposure observed between the villages in the two surveys, were hence not due to a systematic cause, but may be due to causes such as human behaviour, housing quality [42, 43] and proximity to breeding sites [43]. Significant variations in *Anopheles* bite exposure across villages has been reported in areas with considerable altitudinal variations [15, 16].

In our logistic regression analyses, gSG6-P1 seroprevalence was significantly lower among residents from Oria village compared to the other villages while the levels of exposure to *Anopheles* bites were not significantly associated with age, sex, level of education, bed net ownership, use, and impregnation with insecticides, presence of holes and size of holes. As exposure to mosquito bites depend on mosquito and human behaviour, the possible relationship between bed net use and age further explored. There were no statistically significant differences in the use of bed nets between the age groups nor did we observe any statistically significant differences in the seroprevalence by bed net daily use between different the age groups. We did, however, observe a seroprevalence more than twice as high among children 0–5 years of age who used bed nets daily compared to children who did not use bed nets daily. This is probably due to behaviour as younger children are expected to sleep early and more hours and here the effect of bed net use is more apparent compared to the other age groups who probably get exposed before sleeping hours. A few studies conducted in different settings including Senegal, Cameroon and in the Solomon Islands reported findings similar to this study, where they have documented no statistical significant association between exposure to *Anopheles* mosquitoes and age [22, 38, 39], gender or use of anti-mosquito strategies such as LLINs [22]. Contrary to these studies, other studies have reported that levels of anti-gSG6-P1 IgG responses varied significantly by village and age; e.g. a study conducted on the Myanmar-Thailand border and another study conducted in Podor Senegal [17, 26]. The reason for this variation could be the difference in malaria prevalence for instance in the Myanmar-Thailand villages malaria prevalence varied between 2–12% for *P*. *falciparum* and 7–24% for *P*. *vivax* [26] while the prevalence of malaria in the present study was ≤ 0.5%. The higher the risk of malaria the more likely people are to protect themselves and protect the young from mosquito bites [35]. Thus, the difference in malaria prevalence may probably be due to difference in risk of exposure to vector bites and therefore differences in gSG6-P1 IgG responses between the two settings. For Lower Moshi, the lack of difference in exposure by age and other risk factors studied is perhaps eclipsed by the extremely low malaria transmission intensity extrapolated by the prevalence of malaria. Also, the primary malaria vectors are different for the two settings, where in Lower Moshi, we found that *An*. *arabiensis* are the dominant malaria transmitting vectors while *An*. *minimus s*.*l*. and *An*. *maculatus s*.*l*. were the most dominant malaria vectors in Myanmar villages. These differences may account for the different findings in the two studies since these mosquitoes differ in biting habits, where *An*. *arabiensis* are endophagic [31] while *An*. *minimus s*.*l*. and *An*. *maculatus s*.*l*. are exophagic [44]. In addition to that, significant variations in gSG6-P1 seroprevalence have been reported across villages with different transmission intensities influenced by altitude where seroprevalence was found to be high in low altitude villages and low in high altitude villages [15, 16].

### Conclusion

From this study findings, anti-gSG6-P1 IgG is able to distinguish seasonal fluctuations in exposure to *Anopheles* bites and may be able to function as a sensitive tool to detect exposure to *Anopheles* mosquito bites in extremely low malaria transmission settings where other entomological tools are obsolete. However, in settings such as Lower Moshi, further studies accounting for mosquito behaviour, especially how they interact with their host and perhaps larger sample size with more extensive *Anopheles* sampling are warranted to better understand the correlations between this serological marker and abundance of *Anopheles* mosquitoes.

## Supporting information

S1 Table Numbers of Anopheles mosquitoes collected in each village. (XLSX)

S1 Fig Individual gSG6-P1 IgG levels and numbers of Anopheles mosquitoes collected in households, N = 54. (TIF)

S2 Fig gSG6-P1 seroprevalence by bed net daily use between different age groups. (TIF)

S1 Dataset gSG6-P1 evaluation in Lower Moshi. (XLSX)

S1 File Questionnaire English version. (DOCX)

S2 File Questionnaire Swahili version. (DOCX)
