# Supplementary figures and images for: Use of anti-gSG6-P1 IgG as a serological biomarker to assess temporal exposure to Anopheles’ mosquito bites in Lower Moshi
Source: PLoS One. 2021 Oct 27;16(10):e0259131. doi: 10.1371/journal.pone.0259131 (PMC8550589; doi:10.1371/journal.pone.0259131)

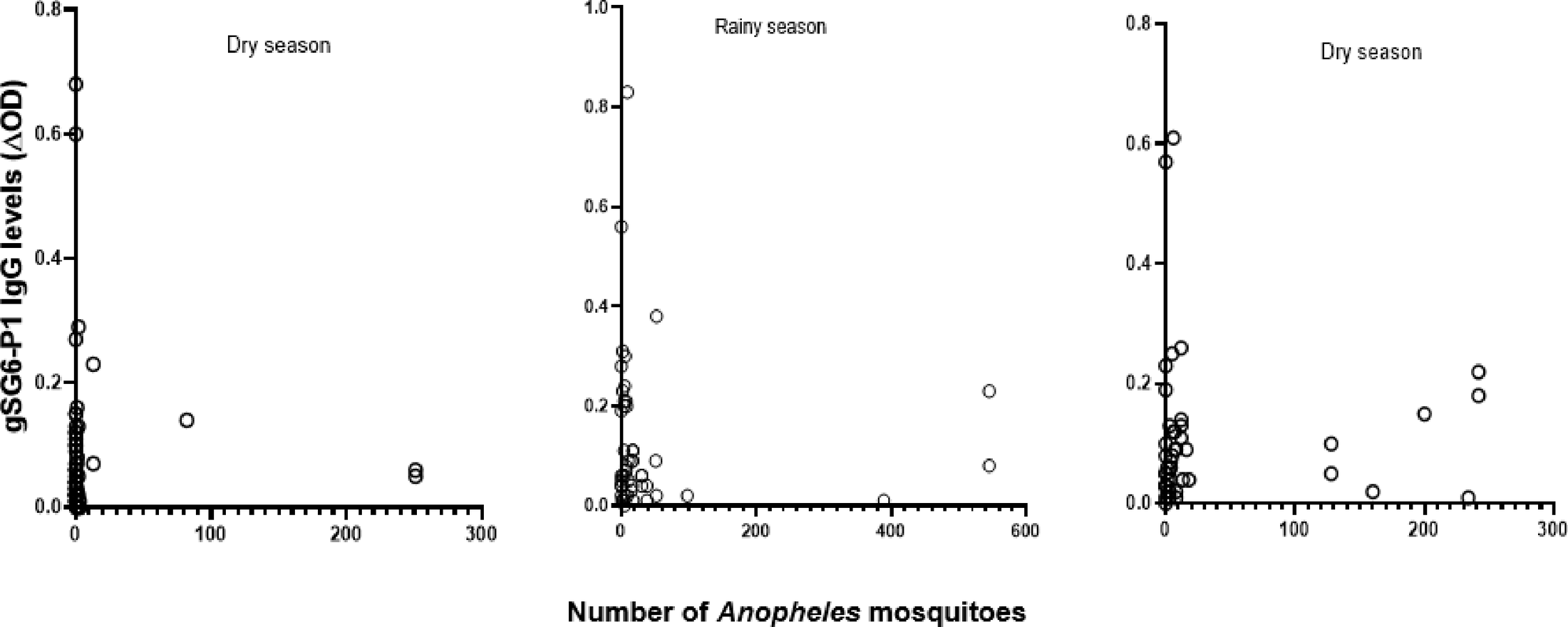

Supplement: S1 Fig — (TIF) [file pone.0259131.s002.tif]

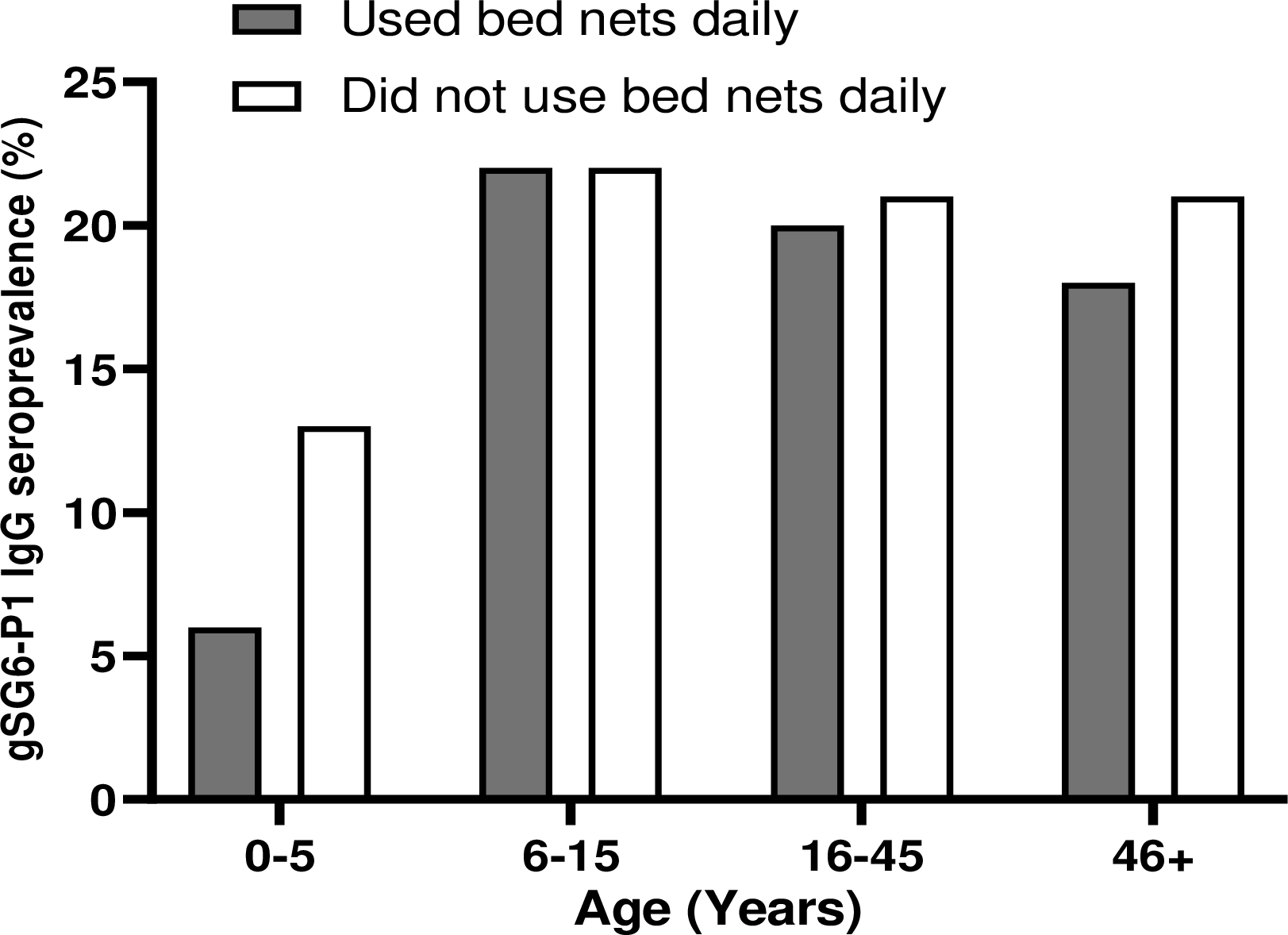

Supplement: S2 Fig — (TIF) [file pone.0259131.s003.tif]
