# Supplementary material for: Use of anti-gSG6-P1 IgG as a serological biomarker to assess temporal exposure to Anopheles’ mosquito bites in Lower Moshi
Source: PLoS One. 2021 Oct 27;16(10):e0259131. doi: 10.1371/journal.pone.0259131 (PMC8550589; doi:10.1371/journal.pone.0259131)
Supplement: S1 File — (DOCX) [file pone.0259131.s005.docx]

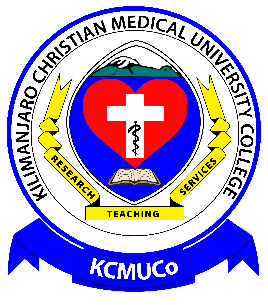


**Kilimanjaro Christian Medical University College, Moshi-Tanzania**

**Building Stronger Universities in Developing Countries**

**SURVEY QUESTIONNAIRE**

**TITLE:** **Use of anti-gSG6-P1 IgG as a serological biomarker to assess temporal exposure to *Anopheles’* mosquito bites in Lower Moshi**

**INVESTIGATORS:** Nancy Kassam, Associate Prof. Reginald Kavishe and Robert Kaaya

**COLABORATORS:** Associate Prof. Michael Alifrangis, Associate Prof. Christian William Wang, Associate Prof. Christentze Schmiegelow

| DATE: ______ / ______ /_________ Participant’s ID Number _______________________  *^(Day / month / year)^*  Initials of interviewer: ___________________ Signature: _______________________  Survey number: _________________ Household identification number _______________ |
| --- |

1. **GENERAL INFORMATION**
   1. Village ______________________________
   2. Household No. ______________________________
   3. GPS coordinates ______________________________
   4. Date of Birth (*day/month/year*) _____ /_____ /______
   5. Participant’s Age _________ Months __________Years

_1 2_

- 1. Participant’s Sex Male Female
  2. Education of a participant

1. No formal education
2. Primary education
3. Secondary education
4. Tertiary education
5. Pupils at primary school (Child)
6.
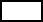
 Child aged < 5 years
7. **BED NET USE & PROTECTION MEASURES**
   1. What is the color of your bed net?

_1 2 3_

Blue White
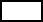
 Other color

1. _2_

2.2 Is your bed net impregnated with insecticides? Yes No

- 1. If yes, what type of insecticide is the bed net impregnated with?

1. Long lasting pre-treated bed net (LLINs)
2. Short lasting individually treated bed net

_1 2_

- 1. Did you/your child sleep under net last night? Yes No

_1 2_

- 1. Is your bed net having holes? Yes No
  2. If yes, what is the size of the largest hole(s)? – see categories on page 6

1. Very small (< 2.5cm)
2. Small (2.5cm)
3. Medium ( 3.6cm)
4. Large ( 18.9 cm)
5. Very large (> 18.9 cm)
   1. List any other thing that is done to protect your household from getting malaria
6. Use mosquito repellent
7. Use smoke to drive away mosquitoes
8. Eat/drink herbs
9. Burn mosquito coils
10. Indoor Spraying
11. Grow certain plants
12. Other (please specify) **_____________________**
    1. Have you travelled outside lower Moshi within the last year? If NO, skip the next question

**_1 2_**

Yes No

- 1. When have you travelled?

1. Two weeks ago
2. One months ago
3. Six months ago
4. One year ago
5. **HISTORY OF ILLNESS**
   1. Have you or your child been ill last two weeks?

**_1 2_**

Yes No

If yes, indicate the main complaints

| - 1. Fever | - 1. Headache |
| --- | --- |
| - 1. Nausea/Vomiting | - 1. Chills |
| - 1. Joint/Waist pains | - 1. Diarrhea |
| - 1. Convulsions | - 1. Jaundice |
| - 1. Fast breathing | - 1. Difficulty in breathing |
| - 1. Pale conjunctivae/palms | - 1. Loss of reactivity |
| - 1. rashes | - 1. Blood in vomitus, urine, or stool; or vaginal or nose bleeding |
| - 1. photophobia (afraid of light) | - 1. Dizziness |
| - 1. Others Specify_____________________________ | |

_1 2_

- 1. If you were ill, did you seek medical care? Yes No

- 1. If yes, what was the diagnosis?

1. Malaria
2. Other illness

_1 2_

- 1. Are you or your child allergic to any drugs Yes No

3.22 If yes, Specify_______________________________________

1. **CURRENT COMPLAINTS**

Do you or your child currently (or in the last 24h) have?

| - 1. Fever | - 1. Headache |
| --- | --- |
| - 1. Nausea/Vomiting | - 1. Chills |
| - 1. Joint/Waist pains | - 1. Diarrhoea |
| - 1. Convulsions | - 1. Jaundice |
| - 1. Fast breathing | - 1. Difficulty in breathing |
| - 1. Pale conjunctivae/palms | - 1. Loss of reactivity |
| - 1. Rashes | - 1. Blood in vomitus, urine, or stool; or vaginal or nose bleeding |
| - 1. Photophobia (afraid of light) | - 1. Dizziness |
| - 1. Others Specify_____________________________ | |

1. **MEASRUEMENT AND TESTS DONE**
   1. Body temperature (tympanic) at visit: _______. ___ °C
   2. RDT (Malaria) _1 2_

Yes No

- 1. Thick and thin blood smears made  _1 2_

Yes N o

1. **SAMPLE TAKEN**
   1. Whole blood sample taken? (~500ul)

_1 2_

Yes No

1. **RESULTS**
   1. Malaria RDT

_1 2_

Positive Negative

18.9 cm

6.3 cm

2.5 cm

Sizes of holes on bed nets according to WHOPES Categories

.

Sizes of holes on bed nets according to WHOPES Categories
