# Supplementary material for: Use of anti-gSG6-P1 IgG as a serological biomarker to assess temporal exposure to Anopheles’ mosquito bites in Lower Moshi
Source: PLoS One. 2021 Oct 27;16(10):e0259131. doi: 10.1371/journal.pone.0259131 (PMC8550589; doi:10.1371/journal.pone.0259131)
Supplement: S2 File — (DOCX) [file pone.0259131.s006.docx]

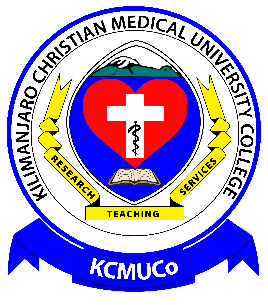


**Kilimanjaro Christian Medical University College, Moshi-Tanzania**

**Building Stronger Universities in Developing Countries**

**DODOSO LA UTAFITI**

**KICHWA:** Matumizi ya anti-gSG6-P1 IgG kama kiashiria-hai cha kiserolojia cha kupima kuwepo hatari ya kuumwa na mbu aina ya *Anofelesi* katika vipindi tofauti eneo la Moshi Chini

**WATAFITI:** Nancy Kassam, Profesa Mshiriki Reginald Kavishe na Robert Kaaya

**WATAFITI WASHIRIKI:** Profesa Mshiriki Michael Alifrangis, Profesa Mshiriki Christian William Wang na Profesa Mshiriki Christentze Schmiegelow

| TAREHE: ______ / ______ /_________ Namba ya Mshiriki _______________________  *^(Siku / Mwezi / Mwaka )^*  Jina la muulizaji kwa ufupi: ___________________ Sahihi: _______________________  Namba ya utafiti: _________________ Namba ya utambulisho wa nyumba ______________ |
| --- |

1. **TAARIFA ZA JUMLA**
   1. Kijiji ______________________________
   2. Nyumba Na. ______________________________
   3. Alama za GPS ______________________________
   4. Tarehe ya kuzaliwa (*Siku/mwezi/mwaka*) _____ /_____ /______
   5. Umri wa mshiriki _________ Miezi __________Miaka

_1 2_

- 1. Jinsia ya mshiriki Kike Kiume
  2. Elimu ya mshiriki

1. Hana elimu rasmi
2. Ana elimu ya msingi
3. Ana elimu ya sekondari
4. Ana elimu ya juu
5. Mwanafunzi wa shule ya msingi (Mtoto)
6.
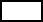
 Mtoto mwenye umri chini ya miaka 5
7. **MATUMIZI YA CHANDARUA & NJIA NYINGINE ZA KUJIKINGA**
   1. Unatumia chandarua cha rangi gani?

_1 2 3_

Bluu Nyeupe
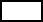
 Rangi nyingine

1. _2_

2.2 Je chandarua chako kimewekwa dawa yoyote? Ndio Hapana

- 1. Kama ndio, ni aina gani ya dawa imewekwa katika chandarua chako?

1. Dawa ya kudumu (LLIN)
2. Dawa ya muda mfupi

_1 2_

- 1. Je, wewe/mtoto wako alitumia chandarua jana? Ndio Hapana

_1 2_

- 1. Je, chandarua chako kina matobo? Ndio Hapana
  2. Kama ndio, tobo kubwa kabisa lina ukubwa gani? – tazama mwongozo uk. 6

1. Dogo sana (< 2.5cm)
2. Dogo (2.5cm)
3. Kiasi (3.6cm)
4. Kubwa (18.9 cm)
5. Kubwa sana (> 18.9 cm)
   1. Njia zipi zaidi ya chandarua unatumia kujikinga usipate malaria?
6. Kupiliza dawa
7. Moshi kufukuza mbu
8. Kula/kunywa mitishamba
9. Kuchoma dawa ya mbu
10. Dawa ya kunyunyiza ndani
11. Kuotesha mimea
12. Nyingine (Taja) **_____________________**
    1. Je, umesafiri nje ya Moshi Chini katika kipindi cha mwaka mmoja? Kama hapana, acha swali linalofuata.

**_1 2_**

Ndio Hapana

- 1. Ulisafiri lini?

1. Wiki mbili zilizopita
2. Mwezi mmoja umepita
3. Miezi sita imepita
4. Mwaka mmoja umeopita
5. **HISTORIA YA UGONJWA**
   1. Je, wewe au mtoto wako mmeugua katika kipindi cha wiki mbili?

**_1 2_**

Ndio Hapana

Kama uliugua, ulipata dalili zipi?

| - 1. Homa | - 1. Maumivu ya kichwa |
| --- | --- |
| - 1. Kichefuchefu na kutapika | - 1. Baridi |
| - 1. Maumivu ya viungo | - 1. Kuharisha |
| - 1. Kifafa | - 1. Manjano |
| - 1. Kupumua haraka | - 1. Kupumua kwa shida |
| - 1. Kupungukiwa damu | - 1. Kushindwa kufanya shughuli |
| - 1. harara | - 1. Damu katika matapishi, choo, mkojo |
| - 1. Kutopenda mwanga | - 1. Kizunguzungu |
| - 1. Nyingine (Taja) _____________________________ | |

_1 2_

- 1. Wakati unaumwa, ulikwenda hospitali? Ndio Hapana

- 1. Kama ulikwenda hospitali, vipimo vilionyesha ugonjwa gani?

1. Malaria
2. Mafonjwa mengine

- 1. Wewe au mtoto wako mnasumbuliwa na matumizi ya dawa yoyote?

_1 2_

Ndio Hapana

3.22 Kama mnasumbuliwa, zitaje_______________________________________

1. **HALI YA KUUGUA KWA SASA**

Je, wewe au mtoto wako mna dalili zozote za kuumwa kwa sasa (au ndani ya saa 24)?

| - 1. Homa | - 1. Maumivu ya kichwa |
| --- | --- |
| - 1. Kichefuchefu na kutapika | - 1. Baridi |
| - 1. Maumivu ya viungo | - 1. Kuharisha |
| - 1. Kifafa | - 1. Manjano |
| - 1. Kupumua haraka | - 1. Kupumua kwa shida |
| - 1. Kupungukiwa damu | - 1. Kushindwa kufanya shughuli |
| - 1. Harara | - 1. Damu katika matapishi, choo, mkojo |
| - 1. Kutopenda mwanga | - 1. Kizunguzungu |
| - 1. Nyingine (Taja) _____________________________ | |

1. **VIPIMO VILIVYOFANYIKA**
   1. Jotoridi la mwili alipotembelewa: _______. ___ °C
   2. RDT (Malaria) _1 2_

Ndio Hapana

- 1. Kipimo cha damu cha malaria kwa darubini _1 2_

Ndio Hapana

1. **SAMPULI ZILIZOCHUKULIWA**
   1. Damu? (~500ul)

_1 2_

Ndio Hapana

1. **MAJIBU YA VIPIMO**
   1. Malaria RDT

_1 2_

Chanya Hasi

2.5 cm

18.9 cm

6.3 cm
